# Development and Characterisation of Antibody-Based Optical Imaging Probes for Inflammatory Bowel Disease

**DOI:** 10.3390/ph14090922

**Published:** 2021-09-13

**Authors:** Matthijs David Linssen, Wouter Tjerk Rudolph Hooghiemstra, Annelies Jorritsma-Smit, Derk Pieter Allersma, Gerard Dijkstra, Wouter Bastiaan Nagengast

**Affiliations:** 1 Department of Gastroenterology and Hepatology, University of Groningen, University Medical Center Groningen, 9700 RB Groningen, The Netherlands; m.d.linssen@umcg.nl (M.D.L.); w.t.r.hooghiemstra@umcg.nl (W.T.R.H.); gerard.dijkstra@umcg.nl (G.D.); 2 Department of Clinical Pharmacy and Pharmacology, University of Groningen, University Medical Center Groningen, 9700 RB Groningen, The Netherlands; annelies.jorritsma@gmail.com (A.J.-S.); d.p.allersma@umcg.nl (D.P.A.)

**Keywords:** fluorescent imaging, monoclonal antibodies, inflammatory bowel diseases, fluorescent molecular endoscopy

## Abstract

Monoclonal antibodies are an important addition to the medicinal treatment paradigm for IBD patients. While effective, these agents show a high degree of primary and secondary non-response, and methods to predict response are highly desired. Information on drug distribution at the target level is often lacking. Fluorescent endoscopic imaging using labelled antibody drugs may provide insight regarding drug distribution, target engagement and drug response, but these assessments require stable and functional fluorescently-conjugated probes. Infliximab, vedolizumab, adalimumab and ustekinumab were conjugated to IRDye 800CW, IRDye 680LT and ZW800-1. The resulting 12 tracer candidates were analysed and characterised on SE-HPLC, SDS-PAGE, iso-electric focussing (IEF) and ELISA in order to evaluate their feasibility as candidate clinical tracers for cGMP development. Major differences in the conjugation results could be seen for each conjugated drug. For Infliximab, 2 conjugates (800CW and 680LT) showed formation of aggregates, while conjugates of all drugs with ZW800-1 showed reduced fluorescent brightness, reduced purification yield and formation of fragments. All 6 of these candidates were considered unfeasible. From the remaining 6, ustekinumab-680LT showed reduced binding to IL23, and was therefore considered unfeasible. Out of 12 potential tracer candidates, 5 were considered feasible for further development: vedolizumab-800CW, vedolizumab-680LT, adalimumab-800CW, adalimumab-680LT and ustekinumab-800CW. Infliximab-680LT and ustekinumab-680LT failed to meet the standards for this panel, but may be rendered feasible if tracer production methods were further optimized.

## 1. Introduction

Ulcerative Colitis (UC) and Crohn’s Disease (CD) are idiopathic relapsing-remitting inflammatory diseases of the gastrointestinal tract, and the two main presenting forms of inflammatory bowel disease (IBD). In Europe, over 2.5 million people suffer from IBD [1]. While the overall mortality of IBD is low, patients experience considerable morbidity and reduction in quality of life. Symptoms mainly include abdominal pain, diarrhoea, bloody stool and cramps, but IBD can also manifest with several inflammatory responses in other parts of the body, such as the joints, eyes, skin or liver, and the disease has been associated with societal burden due to pain, anxiety and depression [2,3,4].

Classically, IBD has been treated with anti-inflammatory agents such as sulfasalazine and mesalazine, glucocorticoids, and immunomodulators such as azathioprine, 6-mercaptopurine or methotrexate [5]. Since the marketing authorization of the monoclonal antibody infliximab in 1998, anti-tumor necrosis factor alpha (TNF-α) therapy has been added to the pharmacological arsenal for treatment of IBD. In the following years, additional new biologicals targeting TNF-α (adalimumab, certolizumab pegol and golimumab) and alternate targets like integrins (natalizumab and vedolizumab) and interleukins (ustekinumab), have been developed [5,6]. The biologicals are currently part of the standard-of-care treatment for the induction and maintenance of remission of moderate to severe UC and CD [7,8,9]. Despite this acceptance into the treatment paradigm, a remaining problem with biological agents is their high rate of both primary and secondary non-response and their high costs. Reasons for the lack or loss of response have not been fully established yet; therefore, better understanding of the mechanism of action and methods for prediction of which patients will respond to treatment are highly desired [9,10,11,12,13].

Optical molecular imaging is a novel technology that has recently seen rapid development in several fields of medicine [14,15,16,17,18,19]. Based on the biochemical properties of the tissue of interest, a molecule with specific targeting properties is functionalized with a near-infrared fluorescent (700–1000 nm) moiety, which allows for specific imaging of the tissue that contains the targeted antigen. In gastroenterology, fluorescence molecular endoscopy has shown promise in both oncologic diseases [20,21,22] and inflammatory conditions [23,24,25,26].

Selection of the optimal therapeutic antibody for the individual IBD patient is currently performed stepwise and requires endoscopic inspection of the gut after weeks to months of treatment in order to determine the degree of response. Tools for early prediction of response are currently lacking. Fluorescently labelled antibodies may be applied for optimizing drug dosing by highlighting the target level in vivo on an individual level, elucidating pharmacodynamics at the inflamed mucosa, and even predicting patient response to biologicals, thus individualizing IBD therapy [23,26]. However, the application of optical imaging has been hampered by the fact that tracer production for clinical trials requires extensive development in order to deliver a tracer molecule that is intact, stably conjugated to the fluorescent dye, with retention of its binding affinity to the desired target, as well as being safe to administer to patients. Additionally, compliance of the production process with Good Manufacturing Practices (GMP) forms a major hurdle for the implementation of optical tracers in clinical practice. Early selection of tracer molecules that are feasible for clinical use could streamline this process.

In this paper, we demonstrate methods for testing and selecting potential antibody-dye conjugates for fluorescent imaging in IBD, and explore the translational feasibility of four antibodies as optical imaging probes for human trials: infliximab, adalimumab, vedolizumab and ustekinumab. We demonstrate the results of conjugating antibodies with multiple near-infrared dyes and a panel of feasibility tests to determine chemical compatibility and show basic characterization of the conjugates.

## 2. Results

### 2.1. Tracer Conjugation and Characterization

We conjugated infliximab, adalimumab, vedolizumab and ustekinumab to IRDye 800CW, IRDye 680LT and ZW800-1 resulting in 12 antibody-dye combinations. Dyes and conjugated products are abbreviated to “800CW”, “680LT” and “ZW800” as abbreviation or suffix, respectively. Size-exclusion high performance liquid chromatography (SE-HPLC) peak shapes of native, buffer exchanged, conjugated and purified antibodies showed that all antibodies were unaffected by the alkaline conditions (pH 8.5) of the conjugation buffer, but incubating with IRDye 800CW or IRDye 680LT resulted in major changes to chromatogram peak shape for infliximab, indicating formation of aggregates (higher-molecular weight protein content). Aggregate peaks overlapped with the monomer peak, making quantification of the level of aggregates not feasible. Conversely, all 4 antibodies incubated with ZW800 showed peak tailing, suggesting antibody breakdown into smaller protein molecules or antibody fragments. In addition, ZW800 conjugates with vedolizumab, infliximab and ustekinumab displayed 15–20% reduced protein yield compared to 800CW-conjugated and 680LT-conjugated antibodies. Adalimumab conjugates do not display such a clear difference, but the ZW800 conjugate still had 5–6% lower yield compared to the other adalimumab tracers. The integrity of the antibody monomer is questionable after conjugation to this dye. Representative examples of chromatography for all antibodies are displayed in Figure S1.

Spectral analysis of the absorbance and fluorescence emission signals of each conjugate showed no noteworthy deviations from the expected absorbance peaks for protein (279 nm) and dye components (800CW: 778 nm; 680LT: 678 nm; ZW800: 772 nm), and fluorescence peaks for each dye (800CW: 800 nm; 680LT: 700 nm; ZW800: 795 nm) in any sample, and results were consistent between different samples. A 3–5 nm shift in peak absorbance could be seen for 800CW and 680LT upon conjugation (see Figure S2).

The percentage of monomer-conjugated dye after incubation (label efficiency) was ≥80.0% in all samples, indicating a robust conjugation process. Besides the previously mentioned overlapping aggregate peaks for infliximab, purity was also high, as no protein aggregates were detected and unconjugated dye was detected at very low levels (<1.4%) in all but one sample: one batch of ustekinumab-ZW800 unexpectedly showed 13.9% unconjugated dye. This outlier is suspected to have been caused by a defect desalting gel column during purification. Full results for all in-process controls, final product analysis, and characterization assays can be found in Table S3.

### 2.2. Tracer Brightness

**Figure 1:**
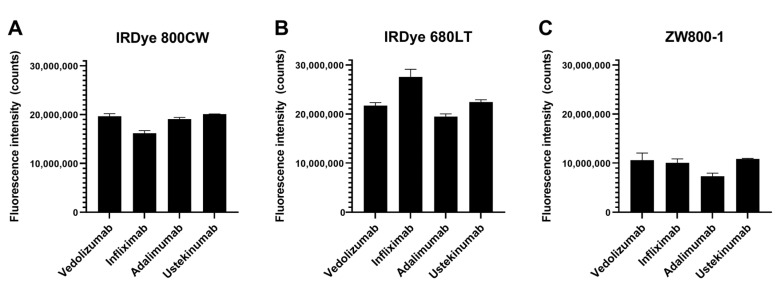
Fluorescent emission of the 12 tracer candidates on Odyssey CLX, grouped by (**A**) IRDye 800CW, (**B**) IRDye 680LT and (**C**) ZW800-1. All antibodies conjugated to 680LT showed fluorescence counts comparable to or higher than 800CW. ZW800 conjugates are approximately half as bright as 800CW conjugates. Higher brightness is a desirable factor for a tracer as a lower dose is required for sufficient signal. Equal aliquots of each tracer batch were measured in a single plate. Data are depicted as the mean and standard error of mean of three tracer batches for each candidate.

Fluorescence brightness measurements (Figure 1) showed 680LT tracers tended to be brighter than 800CW or ZW800 conjugates. Infliximab-680LT is notably the brightest (2.76 × 10^7^ counts), while the other three antibodies conjugated to 680LT show lower signal with comparable brightness between them (range 2.25 × 10^7^–1.95 × 10^7^ counts). Results for these three antibodies were also fairly close in brightness to their 800CW-conjugated equivalents (range 2.01 × 10^7^–1.91 × 10^7^ counts for vedolizumab, ustekinumab and adalimumab), while infliximab was the least bright (1.61 × 10^7^ counts) of the 800CW conjugates. ZW800 conjugates perform worse for all antibodies, showing brightness approximately half that of 800CW conjugates (range 1.09 × 10^7^–0.73 × 10^7^ counts), which could reduce tracer visibility.

### 2.3. SDS-PAGE

**Figure 2:**
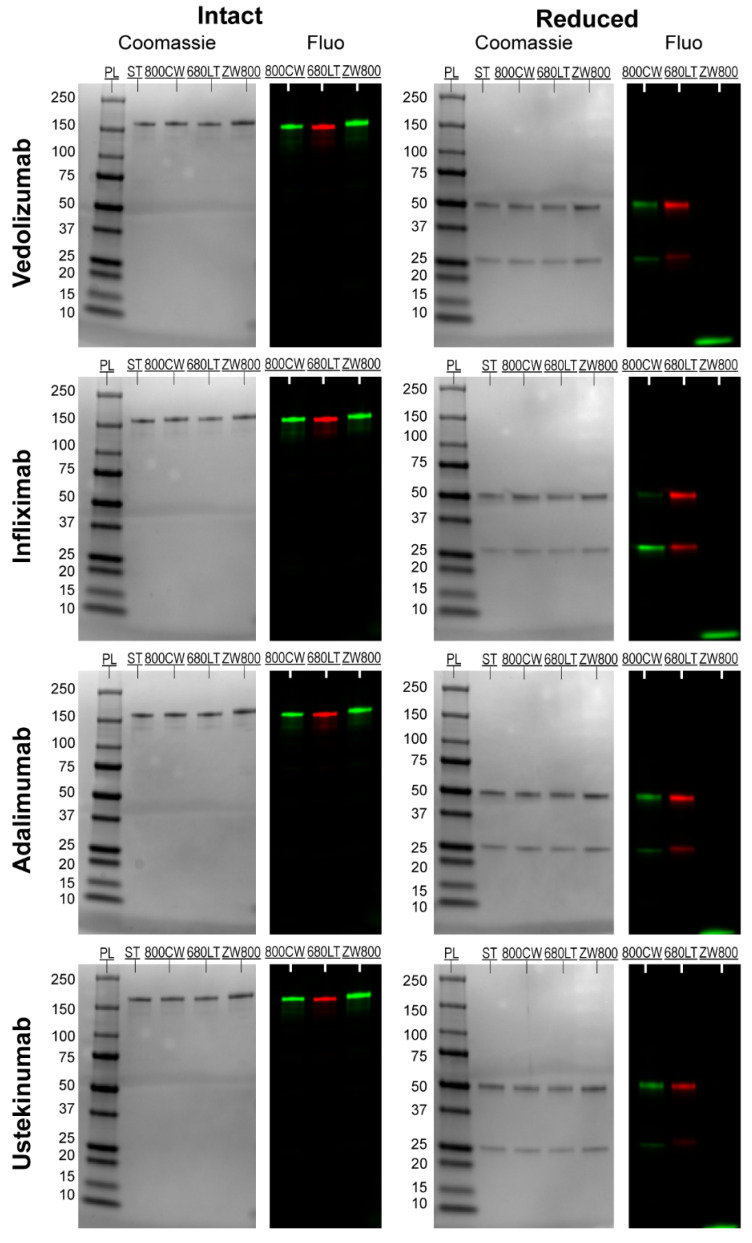
Coomassie-stained white light and Odyssey fluorescence images of sodium dodecyl sulfate polyacrylamide gel electrophoresis (SDS-PAGE) gels. Each image row represents a single antibody. Conjugated samples of all 3 dyes both intact and reduced were run on the same gel both intact and after reduction by β-mercaptoethanol. PL represents the protein ladder, molecular weights for the ladder are depicted left of the coomassie stained bands. Intact antibodies showed clearly defined single bands around 150 kDa, in line with the unmodified reference antibody (ST) lane. Reduced gels showed 2 clearly defined bands, at 50 kDa for heavy chain and 25 kDa for light chain. Fluorescence shows comparable results for all intact antibodies, which show up as clear single bands in their respective fluorescent wavelength (Green: 800 nm, red: 700 nm). After reduction, fluorescent bands can be seen at the 50 kDa and 25 kDa locations for all antibodies, except when conjugated to ZW800. A poorly defined band can be observed at the very bottom of the gel for lanes of reduced ZW800 tracers, while negligible signal is detectable at the reduced band locations. Abbreviations: Fluo: Odyssey CLX fluorescence scan; PL: protein ladder; ST: unconjugated protein reference; 800CW: lane with antibody conjugated to IRDye 800CW; 680LT: lane with antibody conjugated to IRDye 680LT; ZW800: lane with antibody conjugated to ZW800-1.

Coomassie-stained sodium dodecyl sulfate polyacrylamide gel electrophoresis (SDS-PAGE, Figure 2) showed all intact antibodies as well-defined, single bands slightly above the 150 kDa standard and all reduced antibodies as two clearly defined bands around 50 and 25 kDa, as expected. Calculated protein weights were mostly similar for reference antibody and tracers, though increases in tracer weight of up to 10.6 kDa were seen for some intact samples (adalimumab-ZW800 + 9.8, ustekinumab-ZW800 + 10.8). These observations were considered related to the resolution of the gel electrophoresis. Mean calculated molecular weights of all bands are shown in Table S4.

**Figure 3:**
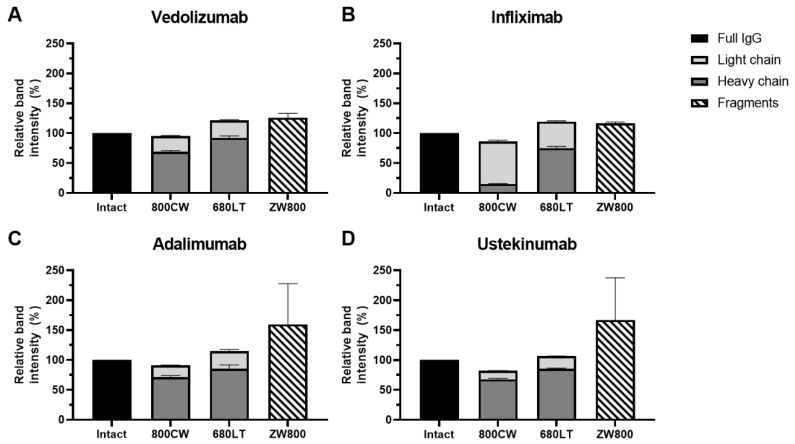
Fluorescent intensity sum of heavy and light protein chains compared to the intensity of the intact antibody (IgG), on sodium dodecyl sulfate polyacrylamide gel electrophoresis (SDS-PAGE). In all antibodies, the sum of mean fluorescent signals on heavy and light chains is comparable to full antibody when conjugated to 800CW (range: 82.0–95.4%) and 680LT (range: 106.6–121.5%). ZW800 conjugates do not show any relevant amounts of fluorescence on the reduced chains, instead a diffuse band at the fluid front of the gel can be seen that matches or exceed the fluorescence intensity of the intact IgG (125.7% (**A**), 116.5% (**B**), 159.8% (**C**) and 167.2% (**D**)). A sum of the heavy and light chain fluorescence signal over 100% of the intact antibody suggests the occurrence of fluorescence quenching in the intact molecules. Data are displayed as the mean and standard error of mean of three measurements.

Fluorescent scans (Figure 2) of the gels showed clear signals for all intact antibodies in their respective wavelength. Bright fluorescent signal was seen on the reduced bands as well for 800CW and 680LT. For ZW800-1, despite the clear bands for heavy and light chain on coomassie, no fluorescence could be seen for reduced samples. Instead, the reduced ZW800-1 samples for each antibody showed a diffuse signal at the very bottom of the gel, near the fluid front. These diffuse bands are represented as “Fragments” in Figure 3.

Vedolizumab, adalimumab and ustekinumab showed fluorescent signal primarily on the heavy chain (HC) of the reduced samples, while infliximab had considerably more conjugated dye on the light chain (LC), especially for 800CW (Figure 3). Combined intensity of reduced chains for 800CW and 680LT was similar to that of intact antibody (range: 82.0–121.5%). However reduced fluorescent bands for ZW800-1 samples (based on location on coomassie) showed <0.20% of the intact antibody signal was on the heavy and light chain, but the “fragments” band showed intensity that was consistently higher than intact antibody (range 116.5–167.2%), suggesting release of all dye from the protein backbone. A sum of the heavy and light chain fluorescence signal over 100% of the intact antibody suggests the occurrence of fluorescence quenching in the intact molecules.

**Figure 4:**
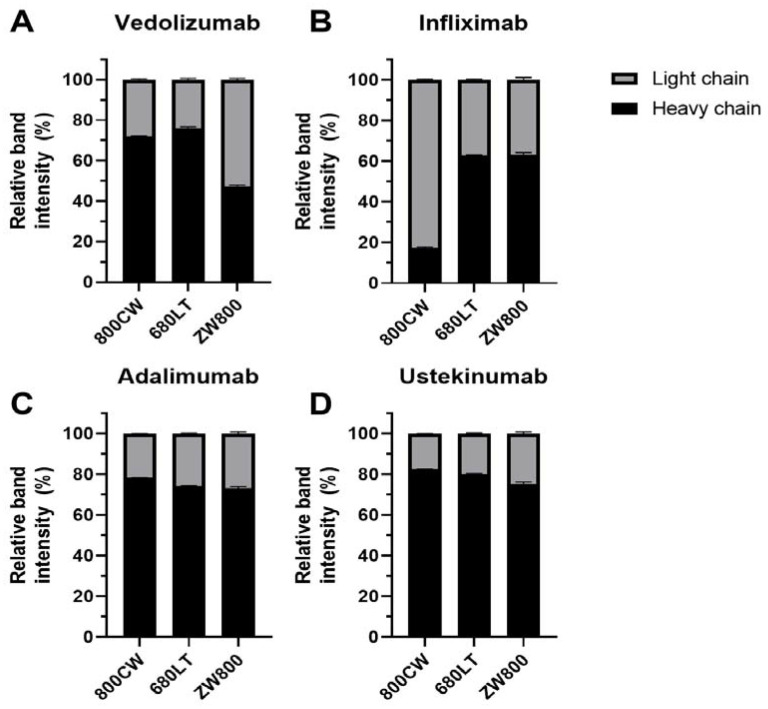
Relative distribution of dye signals across heavy and light chains measured on sodium dodecyl sulfate polyacrylamide gel electrophoresis (SDS-PAGE). ZW800 conjugates did not have visible heavy and light chain signal, data displayed from regions of interest placed at the estimated band position based on coomassie. Adalimumab (**C**) and ustekinumab (**D**) show similar distribution patterns, where dye is mostly on the heavy chain (range adalimumab: 73.1–78.2%, ustekinumab 75.3–82.5%). Infliximab-800CW (**B**) shows a very low amount of heavy chain binding (17.4%). Light chain binding is also seen to a lesser extent in other infliximab conjugates (680LT: 62.8% HC; ZW800: 63.1% HC). Vedolizumab (**A**) shows mostly heavy chain binding, but shows more per-dye fluctuation than other antibodies (800CW: 72.0% HC; 680LT: 75.9% HC; ZW800-1: 47.3% HC). Increased light chain binding could be related to tracer instability.

Relative dye distributions over heavy and light chain (Figure 4; Fragments excluded from analysis) show distributions for adalimumab and ustekinumab were comparable for all dyes, and similar to vedolizumab-800CW and vedolizumab-680LT (range 72–83% of the dye conjugated to the heavy chain). However a shift towards preferential light chain binding was seen for vedolizumab-ZW800 (47% heavy chain) and infliximab-800CW (17% heavy chain). Infliximab-680LT and infliximab-ZW800 also showed reduced heavy-chain binding, but to a lesser extent (62% and 63% heavy chain, respectively).

### 2.4. Iso-Electric Focussing

**Figure 5:**
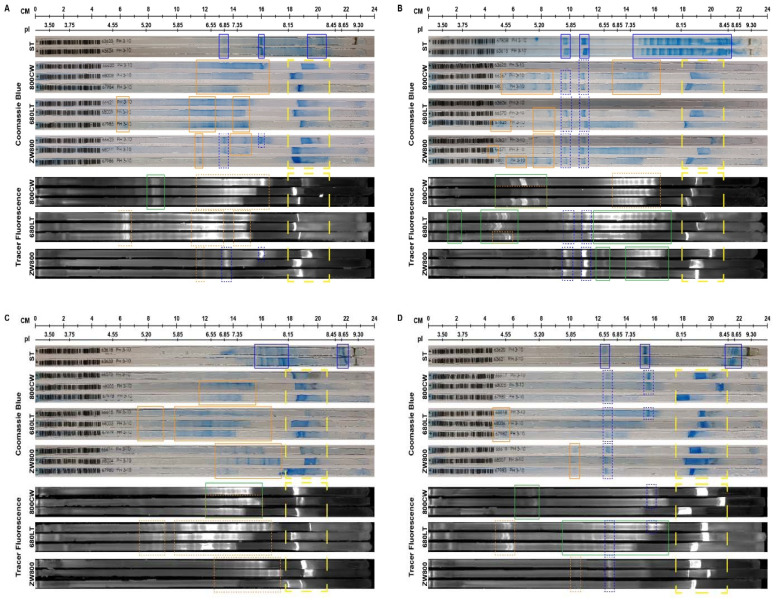
Coomassie blue stain and fluorescent imaging results of completed immobilized pH gradient (IPG) strips. Unmodified reference (ST) and samples from each conjugate (800CW, 680LT, ZW800) were run on separate IPG strips. pI bands and patterns for each reference antibody are highlighted with a solid blue outline. In tracer strips with coomassie, bands that correspond to the reference antibody are given a dotted blue outline, while bands that are seen only after conjugation are given a solid orange outline. In tracer fluorescence, green solid outlines correspond to bands and patterns visible on the fluorescent scans but not on the coomassie-stained strips. Dotted blue and orange outlines correspond to bands marked before with solid blue and orange outlines in reference antibody or coomassie-stained conjugated samples, respectively. The dashed yellow outline highlights areas of the gel that are aspecifically stained as a result of antibody buffer components. Infliximab (**B**) and Ustekinumab (**D**) showed the best retention of their native bands, but all conjugations (vedolizumab (**A**), infliximab (**B**), adalimumab (**C**), ustekinumab (**D**)) resulted in shifts in primary band pI and formation of new bands. Infliximab showed much more acidic bands both natively and especially after conjugation to dyes. Extensive acidic shift may be related to instability in the conjugated protein. The scale of the gels is shown in cm and in pI. pI scale was based on a pI standard ladder (not shown) run on the same system alongside the samples. pI’s of sample bands are interpolated based on the position on the gel and the distribution of pI’s in the pI standard.

Iso-electric focussing of reference antibody and conjugated samples on immobilized pH gradient gel strips (IPG) showed that the observed bands would shift reproducibly for each conjugation, though the extent of changes differed per antibody (Figure 5).

Native vedolizumab showed its main bands at pI 6.85; 7.43 and 8.34. Both 800CW and 680LT show generation of new, less clearly resolved band clusters in the same areas (pI 6.11–7.69), which also display fluorescence. 680LT additionally shows a cluster of 3 highly fluorescent bands around pI 4.81. ZW800 retains the original bands and gains a new cluster around 6.22, all of which show fluorescence.

Infliximab reference showed two 3-band sets, narrowly clustered together around 5.85–6.10, which were still visible in all conjugates. The broad pattern from pI 7.35 to 8.45 in the reference, which was lost upon conjugation, is suspected to be caused by saccharose and polysorbate excipients in the infliximab formulation, which are not present in the conjugated samples, and at far lower levels in the other native antibody samples as a result of higher sample dilutions. Infliximab showed the most extensive acidic shift upon dye conjugation, in particular for 800CW (pI 4.55–5.20) and 680LT (pI 4.55, 5.20 and 3.75). These bands also show clear fluorescence.

In contrast, infliximab-ZW800 shows only minimal extra bands, and fluorescence is most intense on the original bands, and on akaline bands (pI 6.55–7.55). This suggests the effect on iso-electric stability of 800CW and 680LT is bigger than that of ZW800, and a larger acidic shift in iso-electric point appears to correlate with reduced integrity during conjugation.

Adalimumab showed main band clusters at pI 7.42–8.15 and 8.65, but neither were found after conjugation, instead being replaced by more acidic bands at pI 6.38–7.43 for 800CW, 4.55–7.69 for 680LT and 6.55–8.10 for ZW800. All newly formed bands carried the fluorescence of the conjugates.

Ustekinumab showed main bands around pI 7.64 and 6.55. The 7.64 band lost intensity in conjugated samples and was only present in 2 out of 3800CW samples and only one 680LT sample. Only several new bands were seen on coomassie, namely a weak stain cluster at pI 5.85 for ZW800 and at 4.55 for 680LT. Fluorescence for 800CW showed extra bands (pI 4.80–5.20) which did not show up on coomassie. For 680LT, a part of the fluorescence was carried by weak bands that were too poorly defined to identify (diffuse coomassie staining was visible), while ZW800 showed fluorescence on both the reference and newly formed bands of the conjugate, displaying the most conserved antibody structure.

Overall, conjugated samples showed wider and less well-defined bands, or showed slight shifts towards the acidic side of the IPG strip. A per-antibody effect appears to be visible in the formation of more extensive bands either on the acidic or basic side, most notably in infliximab, but the contribution of dye varied per antibody. ZW800 tended to retain most of the native bands.

### 2.5. Target Affinity

**Figure 6:**
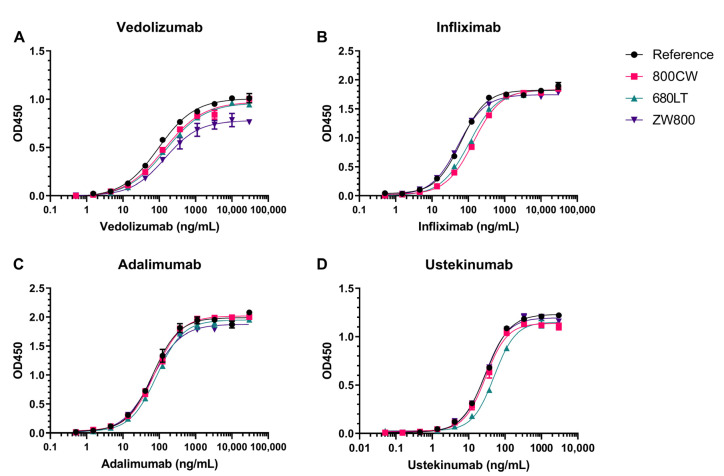
Indirect enzyme-linked immunosorbent assay (ELISA) results for the determination of the affinity of vedolizumab (**A**), infliximab (**B**), adalimumab (**C**) and ustekinumab (**D**) conjugates to their target. Each panel (**A**–**D**) shows overlayed results of one antibody’s reference standard and conjugates of that antibody to 3 different dyes. Serially diluted binding curves were compared to an unmodified reference on the same plate. Affinity was calculated as the ratio between the EC50 values for reference and sample curves. Comparable binding affinities were shown for all conjugates except infliximab-800CW, infliximab-680LT and ustekinumab-680LT

Results from all tracer candidates on indirect enzyme-linked immunosorbent assay (ELISA)-based target affinity assay (Figure 6) show concentration-dependent increase in signal and the characteristic sigmoid curve shape resulting from specific target binding and plate saturation. One point (10,000 ng/mL) in the vedolizumab-800CW curve was excluded from analysis due to high coefficient of variation (>17%) between duplicate values. Target affinity was defined as the quotient of concentration for 50% of maximum intensity (EC50) of reference and tracer sample and expressed as a percentage.

The clearest decrease in antigen binding capacity is seen in the target affinity for infliximab-800CW (45.8%), infliximab-680LT (58.9%) and ustekinumab-680LT (59.1%). The other 9 tracers generally show comparable curves to the reference standard; however, the vedolizumab assay showed some variability in maximum signal measured. Target affinities for conjugates were nevertheless comparable at 71.5%, 63.8% and 66.4% affinity for 800CW, 680LT and ZW800, respectively, showing that the affinity of this antibody is generally impacted similarly by the conjugation of any dye. Infliximab-ZW800 shows close overlap with reference resulting in an affinity of 117.7%. All 3 adalimumab conjugates as well as ustekinumab-800CW and ustekinumab-ZW800 showed close overlap with their reference standards, resulting in target affinities of 77.9–104.7%, 101.2% and 105.4%, respectively. Target affinity values are additionally displayed in Table S3.

### 2.6. Translational Feasibility

**Figure 7:**
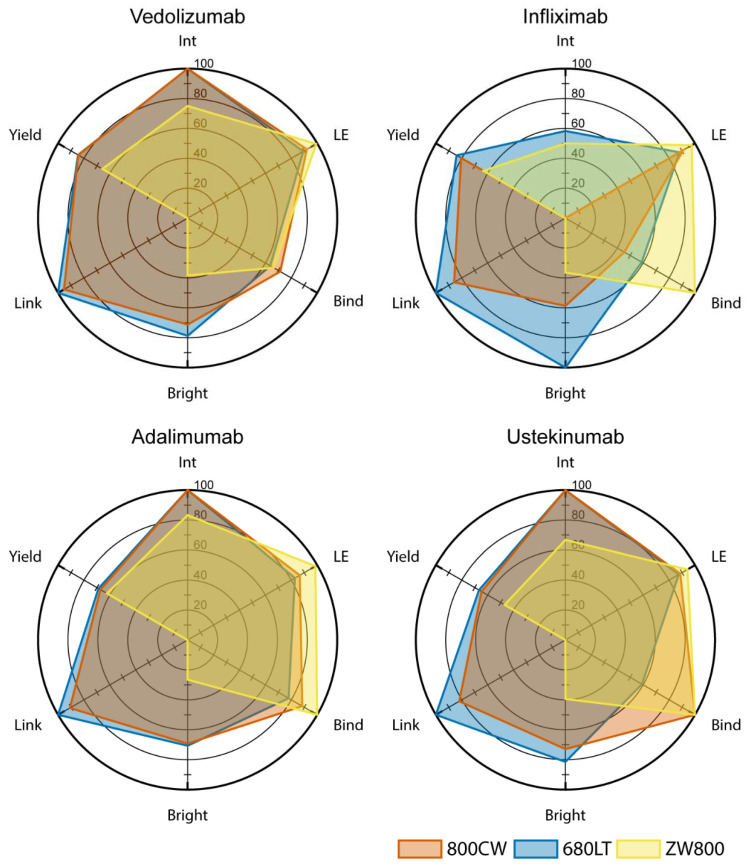
Feasibility assessment radar graphs. A score is calculated in 6 critical parameters for each tracer candidate. Each parameter is scored on a 0–100 scale and plotted on the radar graph for performance comparison within and between antibodies. All six parameter scores were added up for a total feasibility score (Table 1) to determine the most feasible antibody-dye combination. Int: Antibody Integrity; LE: label efficiency; Bind: Target binding affinity; Bright: Fluorescent brightness; Link: Link stability.

To provide a cohesive overview of the results for all tested compounds, we scored the production and characterization results of all 12 tracer candidates on 6 parameters (integrity, label efficiency, target affinity, brightness, link stability and yield) which were considered critical for their feasibility as clinical tracers. Results were plotted in radar charts in Figure 7. Score totals were calculated to determine which of the conjugates showed sufficient feasibility for translation, displayed in Table 1. 

ZW800 conjugates in general underperform in relation to other tracers when it comes to link stability, brightness, protein integrity and yield. All infliximab conjugates have, to some degree, issues with integrity which limits their usability, even though infliximab-680LT was found to have the highest brightness in the panel. Ustekinumab-680LT displayed unexpectedly low target affinity which hampers usability, despite an otherwise promising set of parameters. Taking this into consideration, at this point, vedolizumab-800CW (514.5 pt), vedolizumab-680LT (516.5 pt), adalimumab-800CW (502.5 pt), adalimumab-680LT (500.2 pt) and ustekinumab-800CW (508.3 pt) are all considered feasible for further development and translation.

## 3. Discussion

Ileo-colonoscopy forms the cornerstone for diagnosis, staging and treatment evaluation in IBD. Current endoscopic technologies rely on the expertise of the endoscopist, or on aspecific dyes to evaluate the inflamed areas of the colon [27]. Integrating molecular imaging into the endoscopic routine can enhance and personalize the endoscopy procedure [28,29]. Optical molecular imaging using fluorescently labelled antibodies has been applied in surgical as well as endoscopic settings before, and has shown clear correlation with the antigen being targeted [15,20,21]. To promote the use of targeted imaging in IBD, we tested the feasibility of 12 tracer candidates based on 4 monoclonal antibodies and 3 near-infrared dyes. The tracer molecule is the key component to molecular imaging, and delivering a product fit for clinical trials is resource- and time-intensive, therefore early candidate selection is desired. We performed quality control and characterization assays on all tracer candidates, to evaluate 6 major factors that influence a molecule’s usability in a clinical setting. This resulted, out of the original 12 candidates, in 5 antibody-dye combinations viable for continued development: vedolizumab-800CW, vedolizumab-680LT, adalimumab-800CW, adalimumab-680LT and ustekinumab-800CW. These five tracer candidates display a combination of properties that make them feasible for further development and technology transfer to GMP-compliant environment, and use in (Phase I) clinical trials.

Many properties of the conjugated molecule that contribute to the overall tracer feasibility can be measured or even optimized. A tracer should balance its optical properties with the structural integrity of its protein backbone, to achieve optimal visibility while retaining the pharmacokinetics and pharmacodynamics of the original antibody. To facilitate rapid assessment of this balance for novel tracer candidates we defined 6 criteria as critical: integrity, target binding affinity, and yield to as measures for the integrity of the antibody backbone, and label efficiency, brightness and link stability to assess the optical properties and stability of the fluorophore on the molecule. We found that integrity was a problem for all conjugates based on infliximab. Higher molecular weight molecules were formed upon conjugation with 800CW or 680LT, while smaller fragments and reduced yield were observed for conjugation with ZW800. This effect of ZW800 was observed in the other antibodies as well. In line with the integrity results, we found reduced target binding for infliximab-800CW and infliximab-680LT, as well as for ustekinumab-680LT. Ustekinumab-680LT did not show major problems on other parameters making this reduced binding an unexpected find.

Evaluation of optical properties showed high label efficiency for all combinations, however ZW800 conjugates displayed both low brightness and problems with link stability after reduction of the antibody to heavy and light chains. 680LT as a group showed higher brightness than the other two dyes, and infliximab-680LT was notably the brightest conjugate in the panel, suggesting 680LT conjugation may yield tracers that are more easily visible in vivo.

We performed additional tests to elucidate what caused an antibody-dye conjugate to show a higher or lower feasibility score. On reduced SDS-PAGE, we observed the loss of signal on separated protein chains for ZW800 conjugates and a fluorescent spot at the fluid front of the gel, despite clear protein bands on imperial stain. While the reducing conditions used for the gel electrophoresis are unlikely to occur in vivo, this does suggest that the link between ZW800 and the antibodies is not as robust as the one for 800CW and 680LT. Gel analysis also showed that conjugates with infliximab showed an increase in percentage of the total dye conjugated to the light chain, whereas the other 3 antibodies conjugated dye mostly on the heavy chain.

Analysis on iso-electric focussing showed that conjugation to any dye changed the iso-electric band distribution, but that the extent of changes differed per antibody and per dye. ZW800 conjugates in general showed slightly better retention of the original bands than other dyes, though adalimumab did not show conservation of the original bands in any conjugate. As ZW800 is engineered to be zwitterionic at physiologic pH, this may be related to its reduced impact on the iso-electric point of the antibodies [30]. Infliximab showed bands that were more acidic (pI 5.85–6.15) compared to other antibodies, and also showed much more extensive shift even further towards the acidic side upon conjugation (down to pI 3.75). The use of reduced SDS-PAGE and IEF may be useful tools in the prediction of tracer stability, but additional testing of feasible and non-feasible tracer candidates will be required to confirm the predictive ability of the methods.

Production of an infliximab tracer proved difficult, mainly due to the instability upon conjugation. We observed differences between the effects of the 3 tested dyes however, suggesting that other dyes may not influence the stability to the extent we saw. In the past, infliximab has been conjugated to a radiolabel through cysteine reduction, which resulted in intact isotope-conjugated antibodies [31]. Thus, different methods of conjugation or different payloads could influence the stability of the tracer. The lack of a “one-size-fits-all” solution in our test panel underlines that tracer development, even when based on an established antibody drug, is an involved process which should be custom-fit to the protein at hand. As such, our study has limitations, as additional, orthogonal characterization methods could have been employed to further elucidate effects of conjugation, and we did not perform extensive optimization of production processes or assays for individual antibodies at this point. Some variables that we could have optimized in our labelling process for each candidate include the dye-to-protein ratio during conjugation, buffer conditions during conjugation and purification, or different sites of conjugation such as thiols or Fc-glycans [31,32]. Application of these alternative methods could especially be beneficial for infliximab-680LT and ustekinumab-680LT, which show beneficial factors but with some problems, which may be resolvable. However, our production methods are based on methods used successfully in the past for multiple antibodies [33], and our chosen panel of analyses allow for quick assessment of several key characteristics of new antibody candidates.

In this panel, we weighted all 6 factors equally, in order to compare overall performance and explore the value of all 4 antibodies and all 3 dyes as tracer material. Other panels could use the same factors but with different evaluation criteria, depending on the situation. For instance, while we consider antibody integrity and target binding affinity to be universally critical for tracers, it may be acceptable to have a tracer that does not conjugate at peak efficiency, is less bright, or has low yield, as long as the achieved parameters fit the study they are used in. Development can then focus on resolving potential problems in the compound, or on optimizing the use of properties that are already favourable, facilitating the progress towards a product fit for phase I study.

A potential application of near-infrared imaging for IBD is gaining insight in which patients might show response to therapy. Both adalimumab and vedolizumab have previously been used for optical imaging in clinical trials, labelled with FITC [23,26]. The antibodies were sprayed on the colon wall, and detected by confocal laser endomicroscopy (CLE). Both tracers highlighted inflammatory cells in the mucosa, and the number of positive cells correlated with response to therapy with that antibody. The use of CLE however limits the detection to small point measurements, while wide-field assessment of colon tissue through a working channel fiber, after either intravenous or topical tracer administration, was shown to be feasible [20].

A second promising application of imaging is in visualizing drug distribution in the tissue of interest, which can not only help in individualizing therapy, but also be an important factor during drug development. Therapeutic drug monitoring is currently mostly based on blood levels, and only rarely on measurements of the affected tissue, which can result in a discrepancy between target saturation drug levels and clinically effective drug dose. This discrepancy was observed for vedolizumab, and leaves the pharmacodynamics of this antibody a point of discussion [34,35]. Optical imaging in vivo could help elucidate the mechanism of action by showing which tissue is affected by the drug both macroscopically and microscopically as well as show within-patient heterogeneity. Drug development could also be assisted by this information, as this would allow drug developers to gain better understanding of the drug’s effect in patients during phase I trials.

## 4. Materials and Methods

### 4.1. Antibodies and Dyes

The antibodies Infliximab (Remicade^®^), adalimumab (Humira^®^), vedolizumab (Entyvio^®^) and ustekinumab (Stelara^®^) were investigated. All antibodies were acquired commercially. Stock solutions were made by dissolving lyophilized antibody in water for injections (B. Braun, Melsungen, Germany), or extracting solution from syringes or pens into Eppendorf cups, as appropriate. All stock solutions were stored refrigerated at 2–8 °C during conjugation experiments. Further details on the antibodies are displayed in Table S1.

IRDye 800CW, IRDye 680LT (both LI-COR Bioscience, Lincoln, NE, USA) and ZW800-1 (Curadel, Marlborough, MA, USA) were selected based on the presence of a peak in their fluorescence spectrum in the 690 nm to 800 nm range and availability as NHS-ester. Chemical details on the used dyes are displayed in Table S2. All dyes were dissolved in anhydrous dimethyl sulfoxide (Merck, Darmstadt, Germany) and stored at −80 °C until use.

### 4.2. Conjugation of Dyes to Antibodies

Protocols for the conjugation of dye to the antibody panel were customized procedures derived from in-house labelling procedures of a clinical tracer, which was described previously [33]. Briefly, antibodies were buffer-exchanged to pH 8.5 conjugation buffer (produced in-house) using PD-10 desalting columns (Cytiva Life Sciences, Marlborough, MA, USA) and then mixed with dye in a molar dye-to-protein ratio of 2:1. The mixture was incubated for 2 h in the dark at room temperature (15–25 °C). Conjugated antibody was purified by PD-10, and simultaneously buffer-exchanged to pH 7.0 formulation buffer (produced in-house). Purified protein solution at 1.0 mg/mL was stored refrigerated (2–8 °C) in the dark in glass vials. Using all 4 antibodies and 3 dyes, we were able to produce and analyse 12 separate tracer molecules. Three independent batches were conjugated and purified for each tracer candidate.

### 4.3. Size-Exclusion High Performance Liquid Chromatography

Protein analysis was performed on a size-exclusion high performance liquid chromatography system with diode-array detector (SE-HPLC-DAD) equipped with a Biosep SEC S3000 column and isocratic elution with phosphate buffered saline (PBS) pH 7.6 without modifier at a flow of 1 mL/min. This system was used for measuring the label efficiency, protein monomer concentration, production yield, antibody integrity and two expected impurities: protein aggregates and unconjugated dye.

### 4.4. Conjugated Antibody Optical Properties

0.5 mg/mL dilutions of all tracer solutions were pipetted into two 96-well plates. A translucent and a black plate were used, and sample layout was kept identical between both plates. Spectra for absorbance and fluorescence were measured in the clear and black plate, respectively, on a BioTek Synergy H4 plate reader (Winooski, VT, USA). Absorbance was measured in the range of 230 nm to 850 nm. Fluorescence measurement range was dependent on the dye (800CW/ZW800: 730–900 nm; 680LT: 630–900 nm). The same translucent plate was imaged on the Odyssey CLX (LI-COR, Lincoln, NE, USA) at 700 nm and 800 nm. Data were corrected for blank (unconjugated) antibody intensity. Spectral peaks (Synergy H4) and fluorescent brightness (Odyssey) were determined for each lot of tracer.

### 4.5. Intact and Reduced SDS-PAGE

A tracer sample from each dye conjugate was run on an SDS-PAGE gel alongside an unconjugated control of the same antibody and a Precision Plus Protein All Blue standard (10–250 kDa). Each sample was run on a Mini protean Tetra cell system both natively and reduced by β-mercaptoethanol side-by-side on the same gel. Whole, unstained gels were imaged on Odyssey CLX (LI-COR, Lincoln, NE, USA) to measure fluorescence. Next, gels were stained with coomassie blue dye (Imperial Protein stain, Thermo Scientific, Rockford, IL, USA) and imaged on the Gel Doc EZ scanner. Instrumentation and reagents for SDS-PAGE were acquired from Bio-Rad (Hercules, CA, USA).

### 4.6. Iso-Electric Focussing

One-dimensional iso-electric focussing (IEF) was performed on an Ettan IPGphor II system using 24 cm Immobiline Drystrip immobilized pH gradient strips (IPG, linear pH 3–10). Reference antibody, all conjugated samples and pI standard marker were measured. All reagents and materials were acquired from Cytiva Life Sciences (Marlborough, MA, USA). Refer to Method S1 in the supplementary material for details on the IEF protocol. Focussed IPG strips were fixed, imaged on Odyssey CLX to detect fluorescence, and stained with coomassie blue to detect protein.

### 4.7. Image Analysis

Coomassie SDS-PAGE images were processed by Image Lab (Bio-rad; Hercules, CA, USA) to automatically detect bands and calculate molecular weight of sample bands based on the protein standard. Images were inspected for the presence of the expected and any extra bands.

Odyssey CLX images were processed in Imagestudio v5.2 (LI-COR, Lincoln, NE, USA). To measure fluorescence brightness, measured in 96-well plate, circular regions of interest (ROI) were manually drawn around relevant wells. For band intensity on gels, rectangular ROIs were assigned to each band using the software’s automatic ROI function. All measurements were corrected for background signal and the size of each ROI.

IPG strips were analysed qualitatively by comparing the Coomassie bands for unconjugated antibody to those for conjugated antibodies on white-light images. Next, Coomassie patterns were also compared to fluorescence bands for each corresponding IPG. General shifts in bands and pattern were determined for each tracer. Refer to Method S2 for more details on IPG strip processing.

### 4.8. Target Affinity

Retention of target binding capability was investigated by performing a custom target affinity assay, grouped per antibody. A single representative batch was chosen for each antibody candidate, in order to demonstrate proof of concept whether binding could be retained after conjugation. The assays were based on the principle of an indirect ELISA. Briefly, a 96-well plate was coated with either recombinant TNF-α (Sino Biologicals Europe, Eschborn, Germany, for infliximab and adalimumab), integrin α4β7 or IL23 (for vedolizumab or ustekinumab, respectively; both antigens R&D systems, Minneapolis, MN, USA). Plates were blocked for 1 h with 1% bovine serum albumin in wash buffer. Serial dilutions (in duplicate) of unconjugated antibody and one conjugated sample per dye were incubated on the blocked plate. Bound antibodies for each dilution series were detected by horseradish peroxidase-conjugated secondary anti-human IgG antibodies. Binding was visualized by addition of 1-step ultra TMB ELISA substrate solution (Thermo Fischer Scientific, Waltham, MA, USA), stopping the reaction by addition of 96% sulfuric acid, and measuring optical density at 450nm for each well. Data were corrected for blank wells containing block buffer as sample. A 4-point logarithmic curve was fit to each series and the concentration for 50% of max response (EC50) was calculated from the fit using Gen5 software package (BioTek, Winooski, VT, USA). Relative target affinity (%) was defined as:

(EC50_reference_/EC50_sample_) × 100 (1)

### 4.9. Translational Feasibility

To support decision making with regard to which tracer(s) showed the most beneficial properties for further development and translation for clinical application, 6 results in the current panel from production and QC were designated as critical: antibody integrity, label efficiency, reaction yield, dye-protein conjugation stability, fluorescent brightness and target affinity.

Antibody integrity was defined by the comparability of the peak shape of the conjugated protein compared to the unconjugated reference solution on SE-HPLC. Integrity was scored as intact (100 points), questionable (50 points) or not intact (0 points) for each produced batch, and the average of three batches was input as the score. Label efficiency describes the percentage of total dye that conjugates to protein during the incubation period. This parameter is calculated as the quotient of intact monomer peak area over total peak area, in an SE-HPLC chromatogram at peak absorption for the dye. Reaction yield describes the potential for high-output production processes for the tracer, and is defined as the percentage of protein in the final purified product compared to the protein used as input for the reaction. Dye-protein conjugation stability describes the robustness of the bond between dye and antibody under stressing conditions, and was based on the combined fluorescent intensity of heavy and light chain on SDS-PAGE compared to intact full IgG, expressed as percentage, which was used as score. Fluorescent brightness describes the potential fluorescence output of the tracer per antibody, and was directly derived from the fluorescent counts measured in purified aliquots of the tracer candidates. Data were normalized based on the highest overall result, and the percentage was used as score. Target affinity describes the capacity of the tracer to bind to its target protein. The target affinity is expressed as a percentage of the binding capacity of an unlabelled reference antibody, based on indirect ELISA assays.

Results for each parameter were converted into a 0–100-point score to provide an overall view of tracer performance. For yield, label efficiency and relative target affinity the percentage result was used as the score. For all factors, any score that exceeded 100 points was scored as 100. Scores were plotted on radar charts for visual comparison of tracer properties. The sum of all 6 factors was used as general indicator to display how feasible this tracer is for further development.

## 5. Conclusions

We conjugated 4 antibodies for IBD to 3 near-infrared fluorescent dyes and evaluated the feasibility of the resulting products for translation to a clinical setting. From this panel, vedolizumab-800CW, vedolizumab-680LT, adalimumab-800CW, adalimumab-680LT and ustekinumab-800CW show the most promise to be translated. Infliximab-based conjugates and all ZW800 conjugates showed formation of aggregates or fragments after conjugation, and are therefore considered not feasible for further development at this point. ZW800 conjugates also showed reduced brightness and reaction yield, which hinders development of an efficient clinical process. Any of the five feasible products from this panel, when fully developed and produced under GMP, may be used for clinical trials to enhance the endoscopic toolset and may contribute to the prediction of response to biological treatment.

## Figures and Tables

**Table 1:** Translational feasibility scores.

Tracer	Int	LE	Bind	Bright	Link	Yield	Total	Feasible?
Vedolizumab-800CW	100	92.0	71.5	71.2	95.4	84.4	514.5	Yes
Vedolizumab-680LT	100	89.3	63.8	78.8	100	84.6	516.5	Yes
Vedolizumab-ZW800	75.0	99.2	66.4	38.4	0.1	65.8	344.9	No
Infliximab-800CW	0.0	91.1	45.5	58.7	85.9	80.5	361.7	No
Infliximab-680LT	58.3	88.0	58.9	100	100	84.0	480.2	Potentially
Infliximab-ZW800	50.0	97.6	100	36.4	0.1	63.3	347.4	No
Adalimumab-800CW	100	86.6	88.5	69.2	90.9	67.3	502.5	Yes
Adalimumab-680LT	100	82.8	77.9	70.7	100	68.8	500.2	Yes
Adalimumab-ZW800	83.3	98.6	100	26.5	0.1	62.3	370.8	No
Ustekinumab-800CW	100	88.7	100	72.9	82.0	64.7	508.3	Yes
Ustekinumab-680LT	100	87.5	59.1	81.5	100	66.7	494.8	Potentially
Ustekinumab-ZW800	66.7	94.2	100	39.4	0.1	47.0	347.3	No

Each feasibility score calculated from 0–100. A score ≥ 500 is considered to correspond to a feasible candidate. Potentially feasible candidates don’t meet the set criteria, but may be optimized in specific areas to become feasible. Int: Antibody Integrity; LE: label efficiency; Bind: Target binding affinity; Bright: Fluorescent brightness; Link: Link stability.

## Data Availability

The data underlying this article are available in the article and in its online supplementary material.

## Supplementary Materials

The following are available online at https://www.mdpi.com/article/10.3390/ph14090922/s1, Method S1: Iso-electric focusing protocol, Method S2: IPG result processing, Table S1: General information about antibodies used for conjugation, Table S2: General information about fluorescent dyes used for conjugation, Table S3: Full tracer conjugation parameters and physico-chemical tracer characterization results, Table S4: Molecular weights of intact and reduced antibodies, Figure S1: Chromatographic results of protein standard stock solutions and conjugated samples, Figure S2: Normalized dye absorbance peaks in free and protein-conjugated state.
