# Supplementary material for: Development and Characterisation of Antibody-Based Optical Imaging Probes for Inflammatory Bowel Disease
Source: Pharmaceuticals (Basel). 2021 Sep 13;14(9):922. doi: 10.3390/ph14090922 (PMC8468533; doi:10.3390/ph14090922)
Supplement: Supplementary file 1 [file pharmaceuticals-14-00922-s001.zip › pharmaceuticals-1362466-supplementary.pdf]

Supplementary material

Methods S1: Iso-electric focussing protocol

One-dimensional iso-electric focussing (IEF) was performed on an Ettan IPGphor II system using 24 cm Immobiline Drystrip immobilized pH gradient (IPG) strips with a linear pH range of 3-10. IEF runs were performed with unlabelled reference antibody, all conjugated samples and a pI standard marker. All reagents and materials were acquired from Cytiva Life Sciences (Marlborough, MA, USA).

IPG strips were rehydrated overnight in sample dilutions made in DeStreak Rehydration solution with 0.5% IPG buffer and covered with Immobiline DryStrip Cover Fluid to prevent fluid evaporation and urea crystallization. IPG strip rehydration was performed in IPGphor strip holders as part of the running protocol. The protocol consisted of rehydration at 60V for 14 hours, followed by a hold step at 500V for 1 hour, a gradient from 500V to 1000V for 1 hour, gradient from 1000V to 8000V for 3 hours, and a final hold step at 8000V for 6 hours and 10 minutes. Total run time for each group of 6 samples was 25 hours and 10 minutes.

Methods S2: IPG result processing

After focussing, gels were washed three times by submerging in water for injections for 5 minutes, to remove cover solution. After washing, strips were fixed by submerging in a solution of 12% trichloroacetic acid and 3.5% sulfosalicylic acid in water and incubating overnight on a shaker. Fluorescence intensity of fixed IPG strips was determined by imaging on Odyssey CLX (LI-COR, Lincoln, NE, USA) and consequently stained in coomassie blue in 20% methanol for 30 minutes. Strips were cleared of excess stain by washing in purified water for 2 minutes, followed by immersion in 10% methanol in water until sufficiently destained. White-light photographs of stained IPG strips were used for analysis.

Table S1. General information about antibodies used for conjugation

Antibody	Brand name	Manufacturer	Target	Licensed for
Infliximab	Remicade®	Janssen Biologics	TNF-α	Moderate to severe or fistulizing Crohn's disease, Ulcerative colitis*
Adalimumab	Humira®	Abbvie	TNF-α	Crohn's disease, Ulcerative colitis*
Vedolizumab	Entyvio®	Takeda Pharma	Integrin α4β7	Crohn's disease, Ulcerative colitis, upon failed anti TNF-α treatment
Ustekinumab	Stelara®	Janssen-Cilag	Interleukin-12/32	Moderately to severely active Crohn's disease or Ulcerative colitis, upon failure of other treatments*

*Indications for the use of infliximab, adalimumab and ustekinumab beyond the scope of IBD have been omitted.

Table S2. General information about fluorescent dyes used for conjugation

Dye	Manufacturer	Excitation peak (nm)*	Emission peak (nm)*	Ext.Coeff. at peak (M-1cm-1)*
IRDye 800CW	LI-COR Bioscience	774	789	240,000
IRDye 680LT	LI-COR Bioscience	676	693	250,000
ZW800-1	Curadel Resvet	770	788	253,900

*optical properties depicted for solution in 1xPBS

Table S3. Full tracer conjugation parameters and physico-chemical tracer characterization results

	Vedolizumab			Infliximab			Adalimumab			Ustekinumab		
	800CW	680LT	ZW800	800CW	680LT	ZW800	800CW	680LT	ZW800	800CW	680LT	ZW800
Integrity after buffer exchange	Intact	Intact	Intact	Intact	Intact	Intact	Intact	Intact	Intact	Intact	Intact	Intact
Integrity after conjugation	Intact	Intact	Questionable	Aggregated	Questionable	Questionable	Intact	Intact	Questionable	Intact	Intact	Questionable
Mean label efficiency (SEM)	92.0% (2.124)	89.3% (1.607)	99.2% (0.568)	91.1% (1.477)	88.0% (1.706)	97.6% (0.776)	86.6% (2.776)	82.8% (1.647)	98.6% (0.408)	88.7% (2.598)	87.5% (1.406)	94.2% (3.768)
Absorption peaks (nm)	279; 778	279; 678	279; 771	279; 779	279; 679	279; 772	279; 779	279; 678	279; 772	279; 778	279; 678	279; 773
Emission peaks (nm)	800	700	795	800	700	795	800	700	795	800	700	790
Mean Purification Yield (SEM)	84,4% (2.449)	84.6% (2.729)	65.8% (9.302)	80.5% (2.358)	84.0% (0.633)	63.3% (4.297)	67.3% (0.269)	68.8% (0.358)	62.3% (5.059)	64.7% (1.053)	66.7% (2.276)	47.0% (1.328)
Protein Aggregates	N.D.	N.D.	N.D.	N.D.*	N.D.*	N.D.	N.D.	N.D.	N.D.	N.D.	N.D.	N.D.
Unconjugated dye (SEM)†	0.617% (0.061)	0.947% (0.393)	0.802% (0.114)	0.428% (0.026)	0.886% (0.174)	1.248% (0.304)	0.633% (0.122)	1.364% (0.277)	0.619% (0.046)	0.408% (0.093)	0.280% (0.095)	7.216% (3.875)
Purified dye to protein ratio (SEM)	0.820 (0.080)	1.170 (0.097)	0.477 (0.122)	1.048 (0.087)	1.344 (0.061)	0.297 (0.097)	0.937 (0.010)	1.251 (0.039)	0.511 (0.054)	0.932 (0.031)	1.410 (0.071)	0.4262 (0.054)
Target affinity	71.5%	63.8%	66.4%	45.5%	59.0%	117.7%	88.5%	78.0%	104.7%	101.2%	59.1%	105.4%
Mean Final protein conc. (SEM)	1.021 (0.021) mg/mL	1.005 (0.025) mg/mL	1.055 (0.034) mg/mL	1.047 (0.030) mg/mL	1.073 (0.024) mg/mL	1.053 (0.028) mg/mL	1.054 (0.014) mg/mL	1.031 (0.002) mg/mL	1.069 (0.029) mg/mL	1.092 (0.020) mg/mL	1.088 (0.033) mg/mL	1.015 (0.044) mg/mL

Abbreviations: 800CW: IRDye 800CW NHS ester; 680LT: IRDye 680LT NHS ester; ZW800-1: ZW800-1 NHS ester; N.D. not detectable. Label efficiency expressed as the percentage of dye on HPLC that was conjugated to antibody monomer at the end of the incubation period. Protein aggregate levels defined as percentage of total protein in the sample on HPLC that elutes before the main monomer peak. Unconjugated dye defined as percentage of total dye in the sample on HPLC after purification. * These samples showed abnormalities in their chromatogram peak shape, but did not display fully separated peaks for high-molecular weight species like aggregates, therefore, aggregates have been displayed as N.D. † Mean unconjugated dye for each sample is calculated on all available measurement that did not yield a value below the limit of detection.

Table S4. Molecular weights of intact and reduced antibodies

	Reference standard (kDa)	800CW (kDa)	680LT (kDa)	ZW800(kDa)
Vedolizumab IgG (SEM)	165.6 (2.137)	167.3 (2.288)	166.9 (1.590)	171.3 (1.885)
Vedolizumab HC (SEM)	49.2 (0.291)	48.6 (0.441)	48.8 (0.536)	48.2 (0.470)
Vedolizumab LC (SEM)	25.7 (0.067)	25.4 (0.176)	25.4 (0.176)	25.7 (0.176)
Infliximab IgG (SEM)	171.3 (1.885)	170.2 (3.544)	170.3 (3.247)	175.3 (4.102)
Infliximab HC (SEM)	49.8 (0.100)	49.2 (0.200)	49.2 (0.200)	48.9 (0.353)
Infliximab LC (SEM)	26.2 (0.058)	26.0 (0.145)	26.1 (0.120)	26.1 (0.186)
Adalimumab IgG (SEM)	163.8 (1.650)	166.8 (0.913)	167.3 (0.536)	173.6 (0.751)
Adalimumab HC (SEM)	49.1 (0.176)	48.2 (0.252)	47.9 (0.416)	47.4 (0.367)
Adalimumab LC (SEM)	25.0 (0.100)	24.6 (0.145)	24.3 (0.233)	24.4 (0.353)
Ustekinumab IgG (SEM)	170.4 (2.687)	173.8 (1.770)	176.2 (0.981)	181.0 (2.223)
Ustekinumab HC (SEM)	49.5 (0.100)	48.7 (0.240)	48.7 (0.260)	48.5 (0.481)
Ustekinumab LC (SEM)	24.8 (0.088)	24.3 (0.145)	24.4 (0.173)	24.6 (0.265)

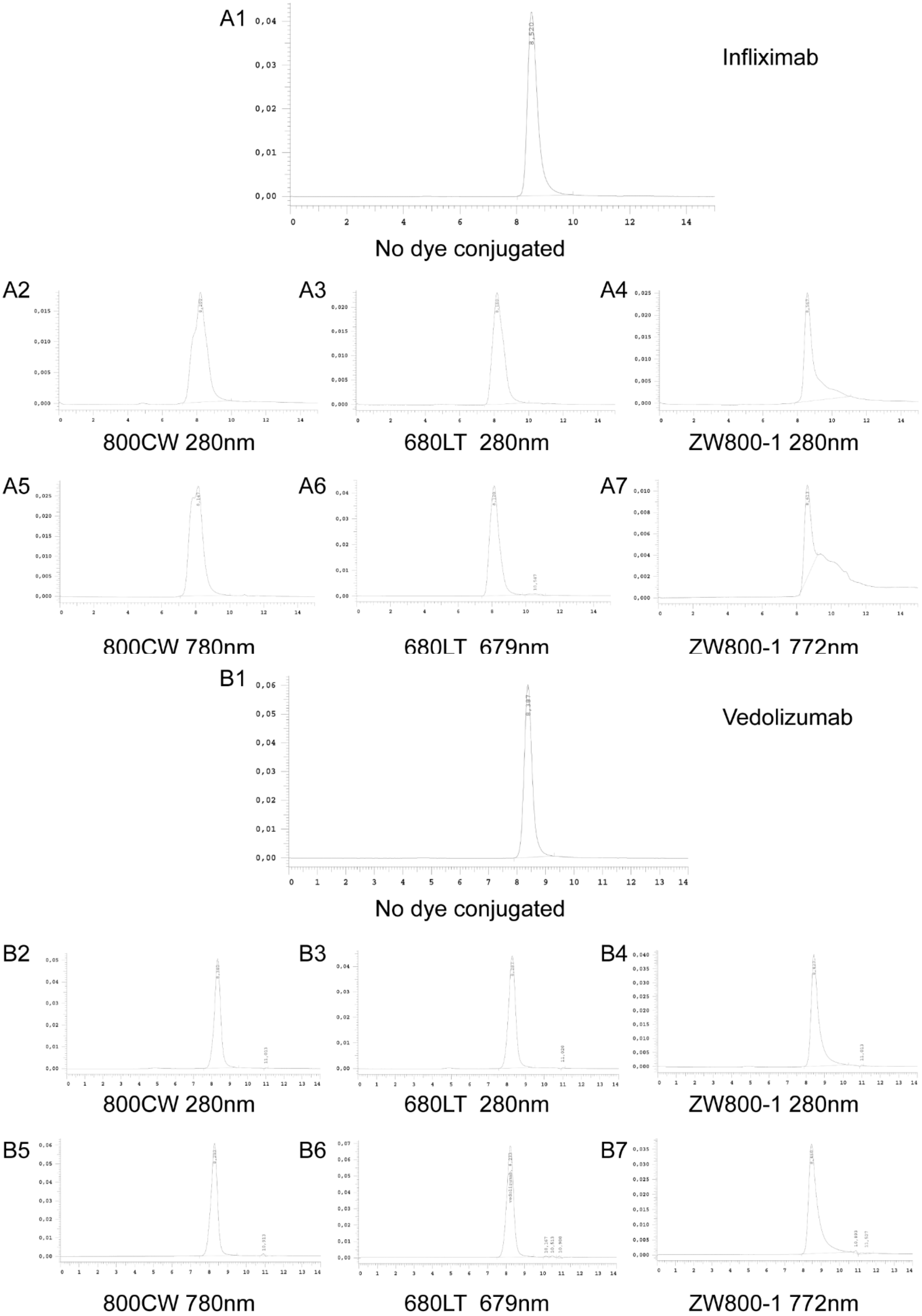
A1
Infliximab
No dye conjugated
A2
800CW 280nm
A3
680LT 280nm
A4
ZW800-1 280nm
A5
800CW 780nm
A6
680LT 679nm
A7
ZW800-1 772nm
B1
Vedolizumab
No dye conjugated
B2
800CW 280nm
B3
680LT 280nm
B4
ZW800-1 280nm
B5
800CW 780nm
B6
680LT 679nm
B7
ZW800-1 772nm

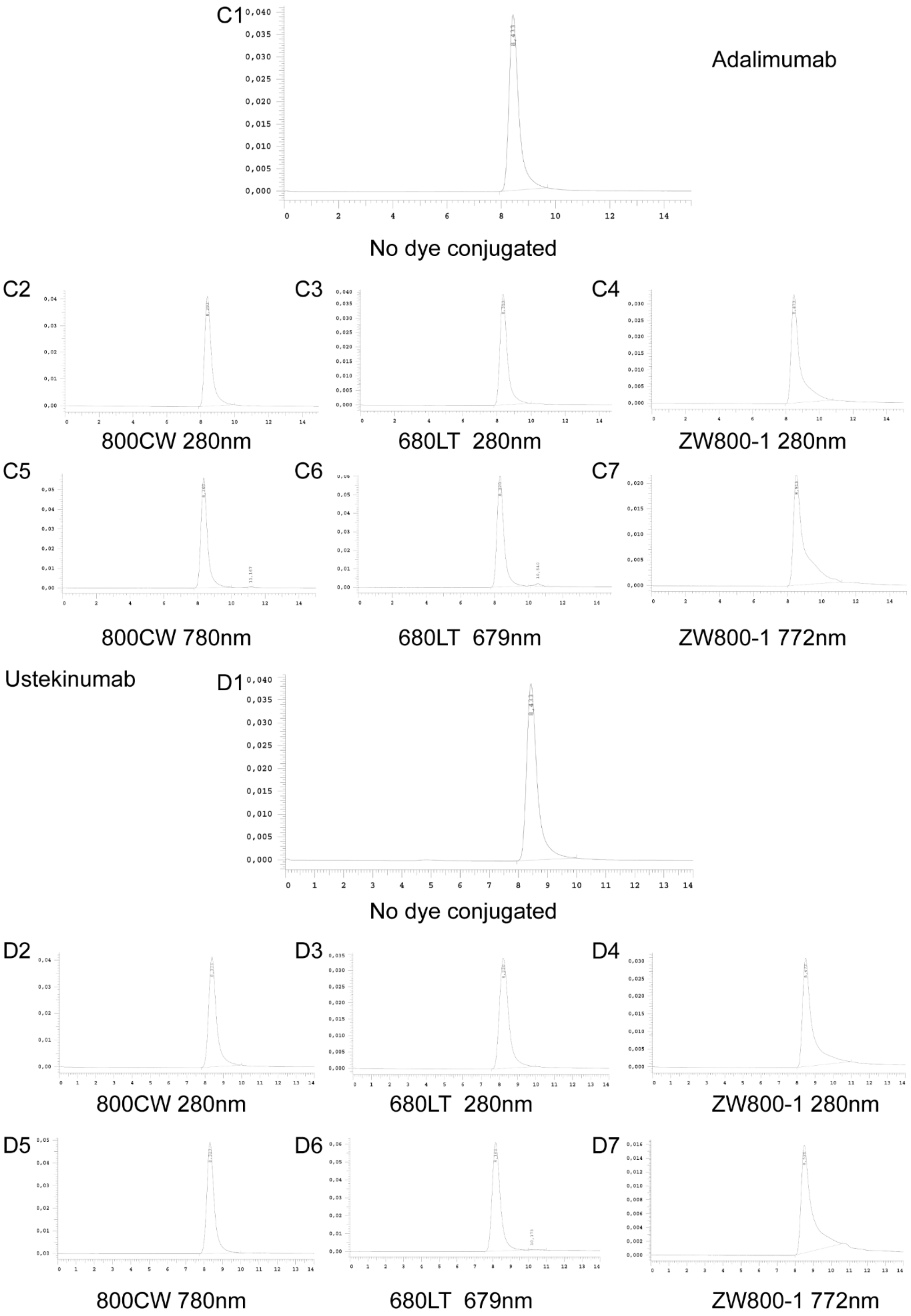

Figure S1. Chromatographic results of protein standard stock solutions and conjugated samples. Each group of panels displays a comparison between the chromatography peak found for unmodified protein and the three conjugated samples, both at 280nm (peptide content) and at the peak absorbance of the dye in the sample. Vedolizumab (**B**), Adalimumab (**C**) and Ustekinumab (**D**) do not show any

significant change in their peak shape upon conjugation to IRDye 800CW or IRDye 680LT. ZW800 shows a notable increase in peak tailing upon conjugation to any antibody (panels 4 and 7 in group **A** – **D**). The tailing is visible in both the 280nm and the 772nm wavelength. Infliximab (**A**) shows formation of additional peaks eluting before the main monomer retention time upon conjugation to both IRDye 800CW (**A2**) and IRDye 680LT(**A3**), suggesting formation of dimers, aggregates, or other high molecular weight protein species. All chromatograms were recorded on a size-exclusion high performance liquid chromatography system with diode-array detection (200 – 900nm).

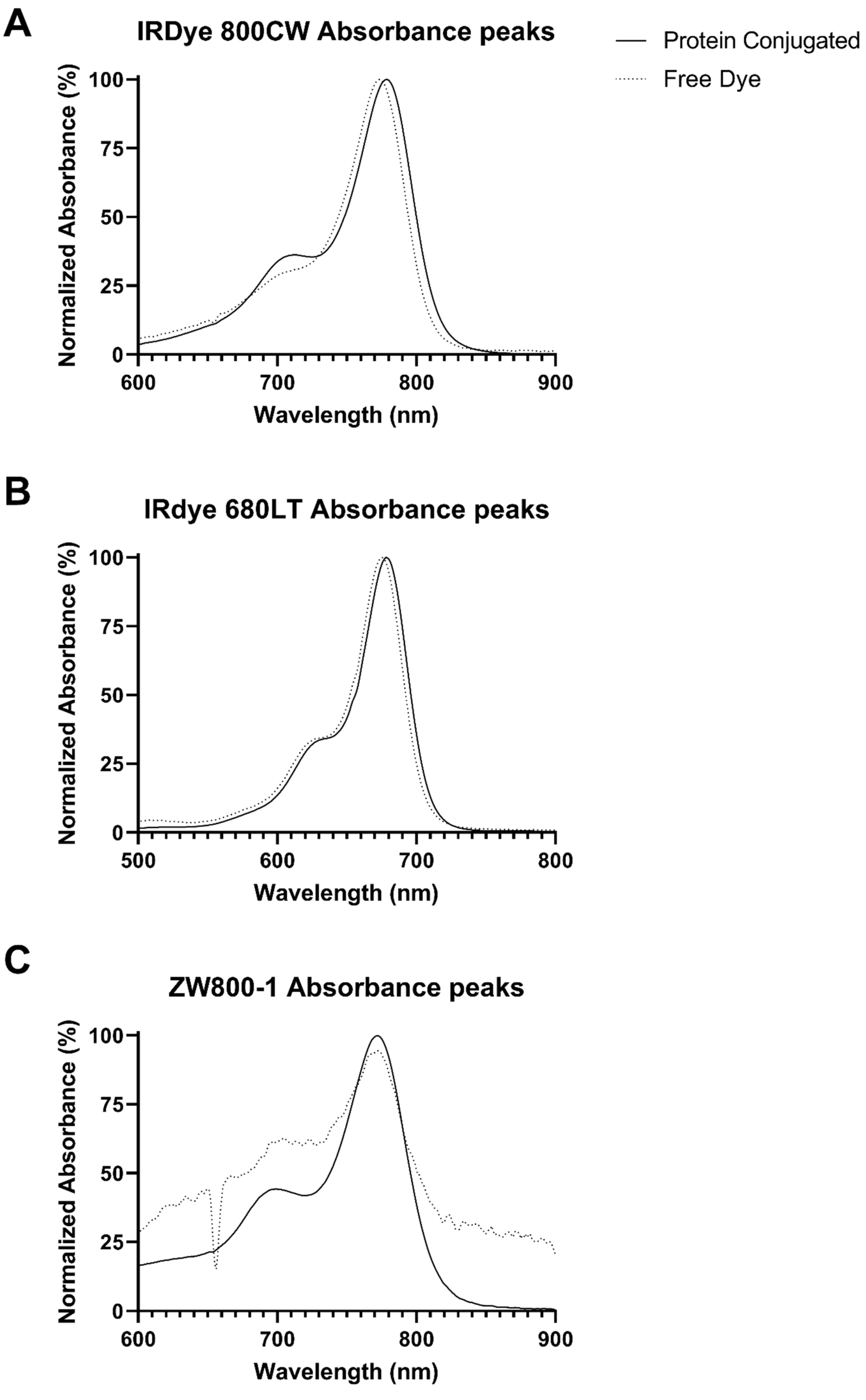
A
IRDye 800CW Absorbance peaks
Protein Conjugated
Free Dye
Normalized Absorbance (%)
100
75
50
25
0
600
700
800
900
Wavelength (nm)
B
IRdye 680LT Absorbance peaks
Normalized Absorbance (%)
100
75
50
25
0
500
600
700
800
Wavelength (nm)
C
ZW800-1 Absorbance peaks
Normalized Absorbance (%)
100
75
50
25
0
600
700
800
900
Wavelength (nm)

Figure S2. Normalized dye absorbance peaks in free and protein-conjugated state. Absorbance for the expected dye wavelengths was captured for all conjugated samples at both the retention peak for the conjugated monomer and the retention peak for unconjugated IRDye. IRDye 800CW (A) and IRDye 680LT (B) show a slight shift (2-5 nm) in absorbance peaks as a result of conjugation of the dye. Absorbance peak for ZW800 (C) was unchanged after conjugation. Peaks for free ZW800-1 were not always well separated from the conjugated peak and their intensity was close to the detection limit of the method, resulting in the noisy signal shown in the graph. Absorbance was measured on diode-array as a part of the SE-HPLC run. Data were normalized against the highest absorbance in the dye spectrum.
